# Reviewed article: Freitas AKMSO, Camargo JDAS, Souza ATB, Pontes THA, Oliveira JMP, Vieira LA, Ferreira PLM, Camargo JF, Freitas JCOC. Sociodemographic and clinical factors associated with delayed diagnosis of cervical cancer: a cross-sectional study, Brazil, 2016- 2020. Epidemiol Serv Saude. 2025:34;e20240764

**DOI:** 10.1590/S2237-96222025v34e20240764.a

**Published:** 2025-09-08

**Authors:** Olinda do Carmo Luiz

**Affiliations:** 1 Universidade de São Paulo, São Paulo, SP, Brazil

## Peer review A

## Questionnaire

Does the manuscript contain new and significant information to justify publication? Yes

Does the Abstract (Summary) clearly and accurately describe the content of the article? No

Is the problem significant and concisely stated? Yes

Are the methods described comprehensively? Yes

Are the interpretations and conclusions justified by the results? No

Is adequate reference made to other work in the field? Yes

## Manuscript Structure

Length of article is: Adequate

Number of tables is: Adequate

Number of figures is: Adequate

Please state any conflict(s) of interest that you have in relation to the review of this paper: None

Interest: Excellent

Quality: Good

Originality: Excellent

Overall: Excellent

Recommendation: Minor Revision

## Comments

O estudo “Fatores sociodemográficos e clínicos associados ao atraso do diagnóstico do câncer de colo uterino no Brasil, 2016-2020”, submetido à revista Epidemiologia e Serviços de Saúde aborda um tema relevante de saúde pública no Brasil, alinhado com as diretrizes da Organização Mundial da Saúde para eliminação do câncer do colo do útero. A análise do impacto socioeconômico no diagnóstico tardio é pertinente e tem potencial subsidiar políticas públicas.

Revisões maiores

O resumo e a discussão dão destaques insuficientes às desigualdades raciais e ao racismo na explicação dos resultados encontrados segundo raça/cor. Sugiro aprofundar o assunto na discussão e dar destaque na conclusão do resumo.

Outro ponto pouco explorado foi o aumento na prevalência de atraso de diagnóstico ao longo do tempo. O texto explica o aumento no ano de 2020 em função da pandemia por Covid19. No entanto, na tabela 3 é possível observar que houve aumento estatisticamente significativo ano a ano desde 2017. Este resultado é muito importante, indica que a atenção ao problema vem piorando, numa franca insuficiência do sistema de saúde.

A diferença quanto a origem do encaminhamento é outro aspecto que poderia ser mais bem discutido. Considere incluir recomendações para novos estudos que estratifiquem o estadiamento, os procedimentos e os óbitos segundo origem do encaminhamento (público e privado).

Revisões menores

Na introdução incluir brevemente uma descrição sobre os exames necessários para a elucidação diagnóstica, bem como uma descrição breve de cada grau do estadiamento, seus significados, prognósticos e recursos diagnósticos. Essas informações ajudarão a interpretar e discutir melhor o resultado de menor prevalência de atraso com maior estadiamento.

Na metodologia a população de estudo não pode ser composta por casos. Reescreva como: “A população de estudo foi composta por pessoas com neoplasia maligna do colo do útero...”

Na seção Fontes de dados descrever mais detalhes sobre a abrangência e cobertura do Registro de câncer do INCA. Sua cobertura não é universal, o que não desmerece a importância do manuscrito.

Na página 6 linha 16, explicar melhor o que significa a frase “Além disso, outro fator que pode estar associado a esses resultados são os parâmetros do perfil sociodemográfico da região, inferior às demais regiões do país” (se preferir, a frase pode ser excluída, já que o modelo ajustou pelo sociodemográfico)

Página 10 - Ilustrações. Faltou o motivo para redução do número de casos de 92.960 para 67.217

